# Delivery of triptolide: a combination of traditional Chinese medicine and nanomedicine

**DOI:** 10.1186/s12951-022-01389-7

**Published:** 2022-04-20

**Authors:** Rui Sun, Jingyue Dai, Mingjian Ling, Ling Yu, Zhiqiang Yu, Longguang Tang

**Affiliations:** 1 School of Pharmaceutical Sciences, Guangdong Provincial Key Laboratory of New Drug Screening, Southern Medical University, Guangzhou, 510515 China; 2 The People’s Hospital of Gaozhou, Maoming, 525200 China; 3 Department of Radiology, Jiangsu Key Laboratory of Molecular and Functional Imaging, Zhongda Hospital, Medical School, Southeast University, Nanjing, 210009, China; 4 Second Clinical College, Guangzhou University of Chinese Medicine, Guangzhou, 510120, China

**Keywords:** Triptolide, Traditional Chinese medicine, Nanomedicine, Passive targeting, Active targeting, Stimuli-responsive targeting

## Abstract

As a natural product with various biological activities, triptolide (TP) has been reported in anti-inflammatory, anti-tumor and anti-autoimmune studies. However, the narrow therapeutic window, poor water solubility, and fast metabolism limit its wide clinical application. To reduce its adverse effects and enhance its efficacy, research and design of targeted drug delivery systems (TDDS) based on nanomaterials is one of the most viable strategies at present. This review summarizes the reports and studies of TDDS combined with TP in recent years, including passive and active targeting of drug delivery systems, and specific delivery system strategies such as polymeric micelles, solid lipid nanoparticles, liposomes, and stimulus-responsive polymer nanoparticles. The reviewed literature presented herein indicates that TDDS is a multifunctional and efficient method for the delivery of TP. In addition, the advantages and disadvantages of TDDS are sorted out, aiming to provide reference for the combination of traditional Chinese medicine and advanced nano drug delivery systems (NDDS) in the future.

**Graphical Abstract:**

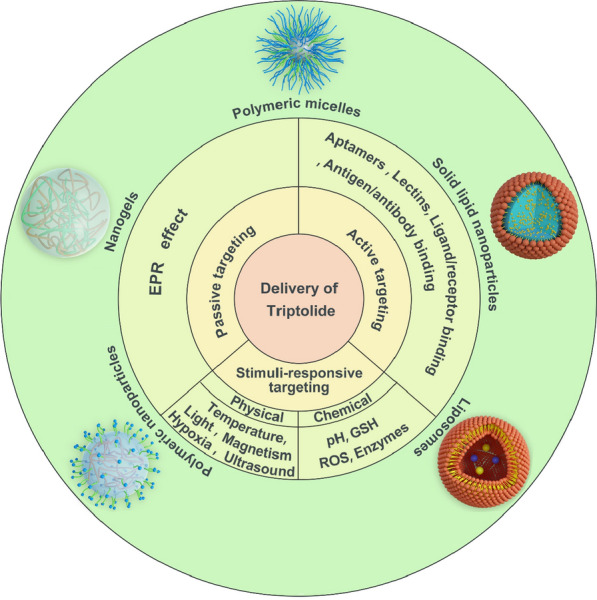

## Introduction

**Fig. 1:**
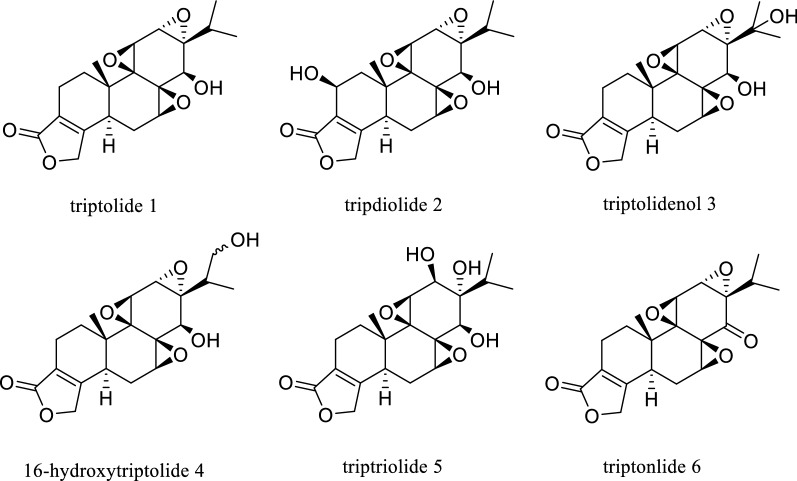
Triptolide and other compounds isolated from TWHF

As one of the core components of traditional Chinese medicine (TCM), Chinese herbal medicine (CHM) has a long history in China. Under the theoretical guidance of TCM, CHM has been applied in clinical practice and has proven to be a treasure for human civilization for thousands of years. During the recent outbreak of coronavirus disease 19 (COVID-19), TCM has been widely used because of its unique advantages and remarkable curative effects against viral infections [1–3]. Among numerous CHMs, triptolide (TP) was considered as the most active epoxide diterpene lactone compound isolated by Kupchan et al. from *Tripterygium wilfordii* Hook F. (TWHF) in 1972 [4]. Its molecular structure is depicted in Fig. 1. TP is also considered as one of the main effective components of TWHF [5].

TP has become a research hotspot because of its highly effective anti-inflammatory, anti-autoimmune, and anti-cancer activities [6–9]. In addition, TP is partly used in the clinical management of rheumatoid arthritis (RA) [10–12]. In recent years, TP has been shown to exert an inhibitory effect on cancer growth, leading to researchers extensively exploring its anti-cancer effects [13–16]. Furthermore, studies have shown that the antitumor mechanism of TP is related to its involvement in the regulation of various molecules and signaling pathways, thereby inhibiting cell proliferation, inducing cell apoptosis, and inhibiting tumor metastasis [15, 16].

**Fig. 2:**
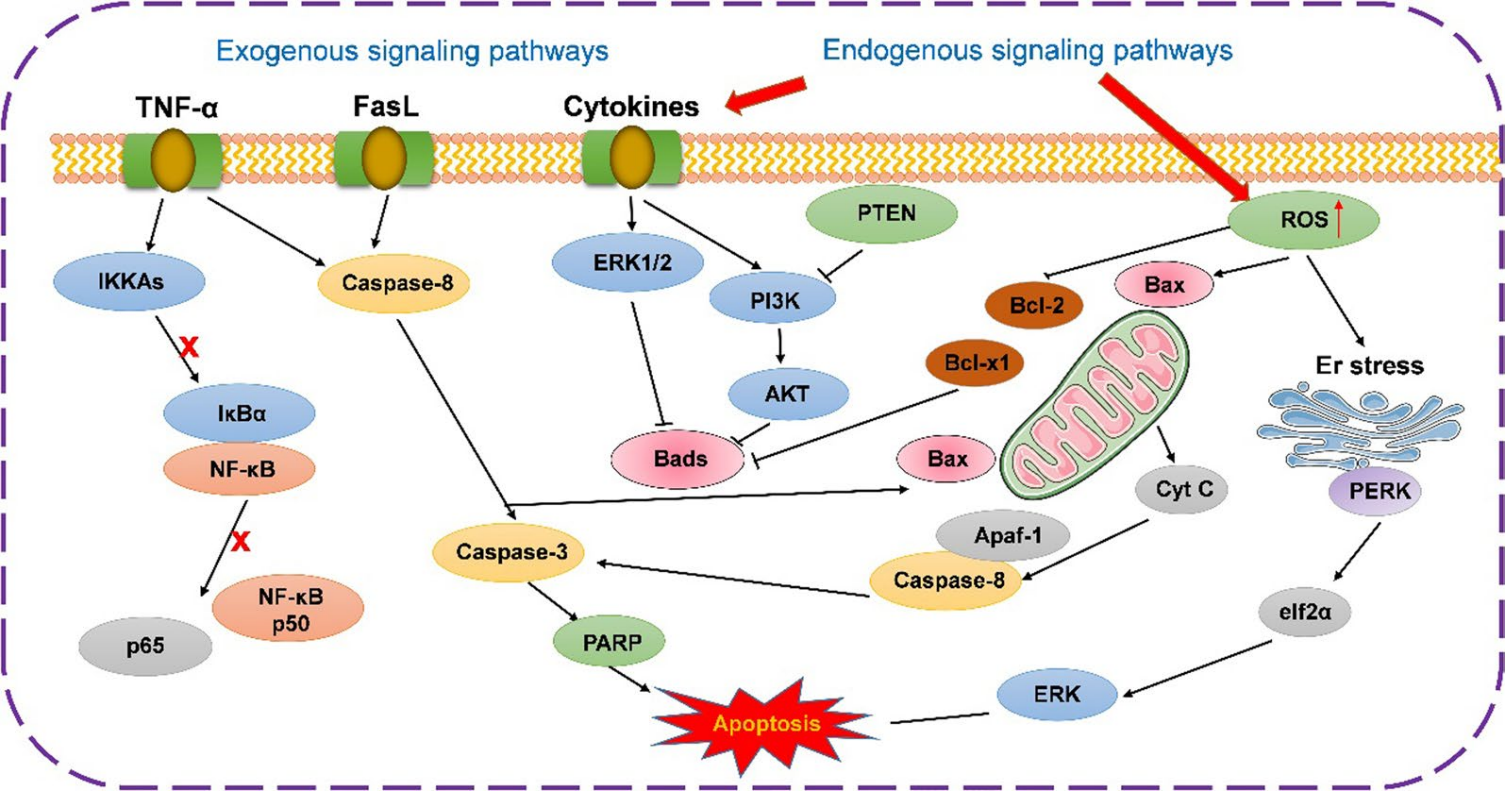
Schematic diagram of tumor apoptosis induced by TP. TNF-α receptor mediated exogenous signaling pathways, mitochondrial and endoplasmic reticulum (Er) stress mediated endogenous signaling pathways. Exogenous signaling pathways: (1) TNF-α—induced apoptosis was enhanced by inhibition of NF-κB. (2) Both TNF-α and FasL simultaneously activated caspase-8/3 signaling pathway and induce apoptosis. Endogenous signaling pathways: (1) By enhancing Bax/Bad and inhibiting the expression of Bcl-2, it further promotes the release of cytochrome C (Cyt C) and mediates apoptosis after activating the Caspase pathway. (2) By disrupting the mitochondrial membrane potential, the release of Cyt C causes oxidative stress on its surface to mediate apoptosis. (3) Er stress induces Er ROS generation, leading to mitochondrial dysfunction and enhanced mitochondrial ROS production

However, the anticancer mechanism of TP is not fully understood. In various known signaling pathways, as those depicted in Fig. 2, [17] the experimental results obtained by different researchers, such as Chang et al., showed that TP further leads to tumor necrosis factor-α (TNF-α)-induced apoptosis by inhibiting NF-κB [13]. In addition, Bing et al. found that caspase-dependent apoptosis of leukemia cells was induced by the mitochondrial pathway at low concentrations of TP [18]. Furthermore, Tan et al. verified that TP-induced extracellular signal-regulated kinase (ERK) activation regulated the expression of the Bcl-2 protein family members, whereas the activation of ERK indicated that excessive reactive oxygen species (ROS) in the endoplasmic reticulum caused oxidative stress and induced apoptosis [19]. It is well known that the invasion and metastasis of cancer complicate the prognosis after surgical treatment. Furthermore, disease progression is related to many factors, such as cathepsin and matrix metalloproteinases (MMPs). Yang demonstrated that TP could reduce the expression of human fibrosarcoma HT-1080 cell matrix metalloproteinase-9 (MMP-9), thereby inhibiting cancer metastasis to a certain extent [20]. In addition, some studies have reported that triptolide can inhibit the expression of interferon-γ (IFN-γ)-induced programmed death-1 ligand-1 (PD-L1) on the surface of tumor cells and reverse the inhibitory effect of tumor cells on CD4+ T cells. It has the potential to target PD-L1 anti-tumor therapy [21, 22]. Gao demonstrated that triptolide has the ability to reshape the immune microenvironment of colon cancer, and its main mechanism is to reduce tumor-associated macrophage infiltration and M2 polarization by inhibiting tumor-derived CXCL12 [23].

Despite being a natural product with a variety of bioactivities, the clinical applications of TP are limited because of its poor water solubility, adverse effects, narrow therapeutic window, and severe toxicity affecting organs and organ systems, such as the liver kidneys, spleen, and the reproductive system [24]. Therefore, it is imperative to design various drug delivery systems including structural modification of the molecule, to effectively deliver TP to the targeted sites of action to decrease the amount of free drugs in other tissues and organs, which will reduce the toxic adverse effects and dosage and enhance its therapeutic efficacy.

**Fig. 3:**
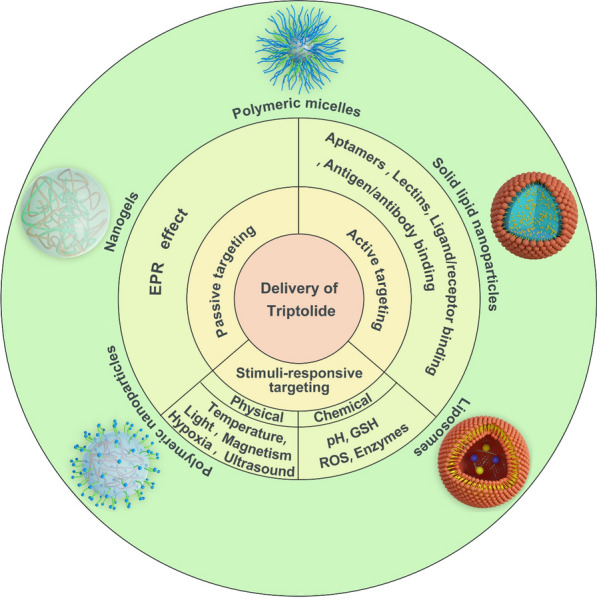
Schematic diagram of triptolide targeted preparation classification

Targeted drug delivery systems (TDDS) are a viable solution to the aforementioned problems. In particular, chemotherapeutic agents cannot be effectively enriched, and most of these drugs distribute to other normal organs throughout the body, leading to serious adverse effects. Nano-drug delivery systems (NDDS), which stem from TDDS, can enrich drugs passively through enhanced permeability and retention (EPR) effects at the tumor site [25, 26]. In addition, more accurate active targeting can make the drug effectively bind to the tumor tissue microenvironment or the specific receptor on the cell surface, thereby achieving a more effective outcome in destroying cancer cells [27, 28]. The two above-mentioned drug targeting methods, viz. passive and active targeting, will be discussed herein. During the past decade, various drug delivery systems have demonstrated remarkable promise for controlled release and targeted drug delivery (Fig. 3), such as polymeric micelles (PMs) [29–32], liposomes [33–36], solid lipid nanoparticles (SLNs) [37], microemulsions [38–40], and polymeric nanoparticles (PNPs) [41–44].

Thus, there is an urgent need to develop NDDS to encapsulate TP, which would likely increase its therapeutic efficiency and reduce its adverse effects. As an ideal drug carrier, it needs to have good biocompatibility, biodegradability [45–47] and avoid triggering the autoimmune defense mechanism of humans or animals [48, 49]. This review focuses on the research and design of targeted delivery systems for TP in recent years, especially in cancer treatment, to provide a reference for further exploration of more intelligent methods to improve the delivery of this molecule and other CHMs and expand the clinical applications of these compounds in the future.

## TDDS for TP

This review classifies and summarizes the recent researches on the combination of TP and nanocarriers according to passive targeting, stimuli-responsive targeting and active targeting. The specific nanocarriers include polymeric micelles, solid lipid nanoparticles, liposomes, polymeric nanoparticles, and nanogels.

### Passive TDDS

#### Polymeric micelle*-*based TP delivery

As a common nano drug delivery system, polymer micelles (PMs) have become a hot spot in the research of anticancer drug delivery because of their advantages of reducing toxicity caused by free drugs, prolonged blood circulation and selectively accumulating at the tumor tissues [50, 51]. These carriers typically have diameters less than 100 nm [52]. PM with a high density of polyethylene glycol (PEG) shells have been shown to avoid recognition by the reticuloendothelial system and preferentially accumulate in solid tumors because of their enhanced EPR effect [51].

Many studies have shown that TP encapsulated in PMs can significantly enhance anti-tumor efficacy compared with free drugs [53]. By detecting thymus index, serum TNF-α and IL-2 levels, Xu et al. not only confirmed that TP- PMs showed no immunosuppressive activity compared with free drugs, but also that the anticancer activity of TP was not weakened after encapsulation. These results indicate that PMs are promising carriers for cancer therapy using TP [54].

As one of the cancers with the highest incidence, 5-fluorouracil has been used as first-line therapy for colorectal cancer in recent decades. However, its side effects and low tumor targeting result in an unanticipated prognosis for patients [55, 56]. Cui et al. designed polymeric micelles loaded with TP derivative LA67 (LA67-PMs) to improve drug accumulation in tumor. In vitro and in vivo experimental results showed that compared with LA67, LA67-PMs not only solved the problem of poor water-solubility of drugs, but also had higher accumulation capacity and good therapeutic effect in tumor cells and tissues by means of EPR effect [57].

Although vascular endothelial growth factor (VEGF) blockers can effectively inhibit angiogenesis, the single target of the drug and the serious toxic side effects caused by the lack of selectivity make it difficult to achieve a good therapeutic effect after administration [58, 59]. According to published in vitro studies, TP inhibits angiogenesis [60, 61]. Using methoxypoly (ethylene glycol)-block poly(ε-caprolactone) as an excipient, Wang et al. encapsulated TP into PMs to prepare a nanoformulation. The pharmacokinetic results indicated that the drug concentration at the tumor site was selectively increased due to the EPR effect of PMs. After TP-PMs treatment, the tumor inhibition rate was increased, and the serum VEGF content was significantly decreased. In addition, immunohistochemical results indicated that the density and diameter of tumor vessels were decreased after TP-PM treatment compared with the control group [62].

Despite the aforementioned results suggesting that TP-PMs has remarkable potential for cancer targeted therapy, can overcome drug toxicity and inhibit angiogenesis, the disadvantages of PMs, such as low drug loading efficiency and poor stability, still require further study and refining.

#### Solid lipid nanoparticle-based TP delivery

Solid lipid nanoparticles (SLNs) are nanocarriers prepared from solid natural or synthetic lipids with particle sizes usually between 50 and 1000 nm. SLNs has been widely studied and applied in recent years due to its characteristics of high drug load, wide applicability, controlled drug release, good biosafety and stability [63–65].

Studies have shown that TP can play a therapeutic effect by significantly enhancing the level of ROS. However, because ROS can damage DNA and induce lipid peroxidation in cells, TP is highly toxic to normal metabolic organs, such as liver and kidney. Mei et al. prepared and characterized tp-loaded SLNs, and observed through a series of experiments that TP-SLNs can effectively reduce liver toxicity while possessing anti-inflammatory activity [64]. The experiment of treating rat foot swelling induced by carrageenan showed that the therapeutic effect of TP-SLNS group was stronger than free TP group. The results of serum physiology and biochemical analysis demonstrated that the hepatotoxicity of the TP-SLNs group was significantly lower than that of the free TP group. The above results indicated that TP encapsulated by SLNs had a good effect of toxicity reducing and efficacy enhancing.

To better elucidate SLNs-based therapy, TP-SLNs were also prepared by Xue et al. The in vivo behavior of SLNs was investigated by tracking and comparing the tissue distribution of free drug and nanoparticles in rats [66]. Through the data analysis of toxicokinetics and tissue distribution results, it was found that the nanoformulation could promote the absorption of TP and control drug release, indicating that one of the reasons for the enhanced efficacy of the nanoformulation may be the change of toxicokinetics. Although SLNs are suitable for drug administration in many ways and have a wide range of drug adaptability, the problems of low drug loading, easy formation of supercooled melts, and drug precipitation must be solved. More specifically, it remains to be studied whether the excipients of SLN affect the efficacy of TP [64, 67]. In addition, after oral administration, SLN is mainly excreted with feces by adhering to the mucosa. However, it should be noted that the particle size of nanoparticles and the characteristics of excipients will directly affect its metabolism and excretion process [68, 69].

#### Liposome-based TP delivery

As one of the most mature nanodelivery carriers, liposomes are one of the few nanoformulation that have been applied in clinical treatment, and their particle size ranges from 50–1000 nm [70]. Due to the special lipid bilayer structure, the water-soluble drugs can be encapsulated in the core and the lipid-soluble drugs can be encapsulated between the lipid bilayers [71–73]. The properties of liposomes, such as amphiphilicity, biocompatibility and biodegradation, are very valuable for the delivery of TCM. In addition, these carriers provide improved therapeutic efficacy and safety, increased bioavailability, sustained release, and localized drug delivery [74].

As a new therapeutic method, photosensitizer-based therapy has received extensive attention from researchers in recent years. Photodynamic therapy (PDT) is one of the two main methods of light therapy, which is based on the fact that a photosensitizer in a tumor is irradiated by a specific wavelength of laser light, which produces a large amount of ROS, thereby causing apoptosis [75]. In order to co-administer TP and PDT to achieve the purpose of enhancing the anti-tumor effect. Yu et al. designed a light-activated liposome (TP/Ce6-LP) combining photosensitizers Ce6 and TP to synergistically treat hepatocellular carcinoma (HCC) using the controlled drug release properties of liposomes and photodynamic therapy [76]. The results of anti-tumor activity studies showed that the combination therapy group induced apoptosis by up-regulating the expression of Caspase-3/PARP protein, and had a good therapeutic effect on patient-derived hepatocellular carcinoma xenografts (PDX^HCC^) after irradiation.

**Fig. 4:**
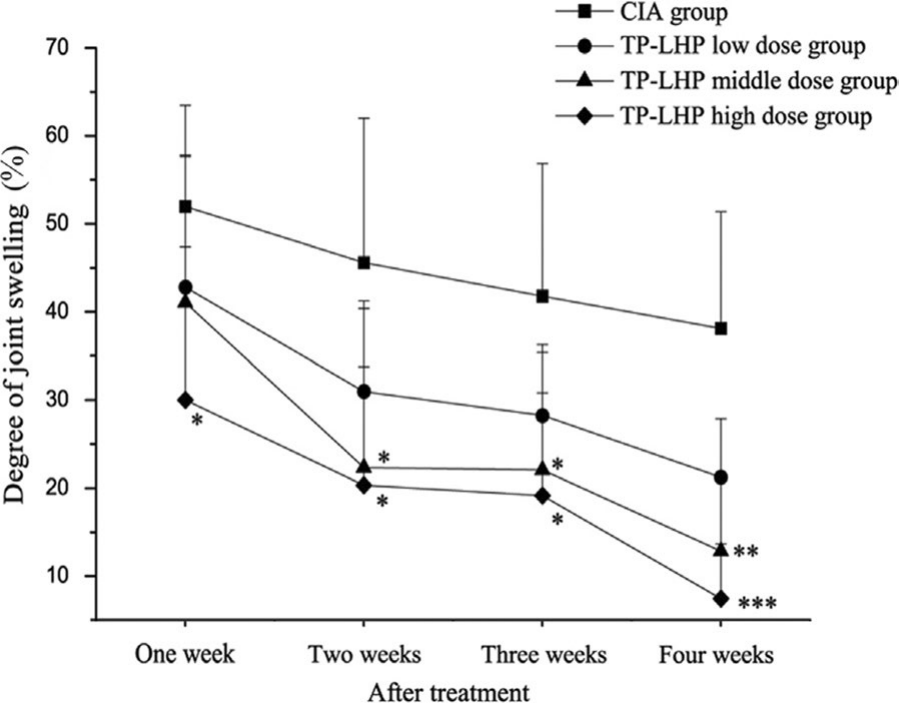
Changes in the degree of joint swelling in the CIA and TP-LHP treated groups. ^*^P < 0.01, ^**^P < 0.01, ^***^P < 0.001 *vs*. CIA group, n = 9. Reproduced with permission from [77]

In the field of TCM, TP is mostly used to treat RA. To improve the efficacy of transdermal drug delivery in collagen-induced arthritis (CIA) rats, Chen et al. prepared a microneedle patch to deliver tp-loaded liposome hydrogel (TP-LHP) for administration, and the experimental results were used to evaluate the pharmacokinetics and pharmacodynamics. All treatment dose groups of TP-LHP could reduce the degree of joint swelling, and the high dose group had the best effect after 1 week of treatment (Fig. 4). Since TP-LHP can continuously and stably release TP, the effect is significant after 4 weeks of continuous treatment. TP-LHP combined with microneedle administration strategy had a good effect in the treatment of RA [77].

In the field of translational medicine, liposomes have attracted more and more attention because of their good stability and biocompatibility. However, their shortcomings, which include low envelopment rate require further improving. In particular, for TCM, compound administration can often play a synergistic role; nevertheless, limited studies on their delivery using liposomes have been carried out. Therefore, the development of liposomes is one of the core contents of TCM pharmaceutics.

#### Polymeric nanoparticle-based TP delivery

Polymer nanoparticles (PNPs) with a particle size range of 10–1000 nm are a kind of carrier prepared by biocompatible and biodegradable polymer [78–80]. PNPs is a promising drug delivery vehicles with a simple manufacturing process that can deliver drugs to specific targets, thereby improving drug safety [81–83]. To increase the therapeutic benefit while minimizing side effects. More and more polymers have been used as excipients for the preparation of nanoparticles, such as poly (lactide-co-glycolic) acid [84], chitosan [85], phosphatidylcholine [86], poly (caprolactone) [87], and carboxymethyl chitosan [88].

Poly (d, l-lactic acid)(PLA) has been widely used in the preparation of nanocarriers for drug delivery systems due to its good biocompatibility and biodegradability, and has been approved by the Food and Drug Administration for clinical application. Liu et al. prepared TP-PNPs with PLA using TP as a model drug, and investigated the renal toxicity of oral administration in rats [89]. In addition, urine samples from 5 groups of rats treated with TP-PNPs and free TP at day 5, 10, and 15 were analyzed. The results showed that compared with free drugs, TP-PNPS could effectively alleviate renal toxicity.

In order to reduce the toxic and side effects of TP during RA treatment, Zhang et al. encapsulated TP into nanoparticles prepared by poly-γ-glutamic acid-grafted l-aspartate di-tert-butyl ester (PAT) [90]. In vivo treatment experiment, nanoparticles accumulated in the inflammatory joint site, and played a good anti-inflammatory effect. In addition, the adverse effect of TNF-α was less than that of free TP. In addition, Liu et al. established a rat model of arthritis induced by complete Freund’s adjuvant, prepared TP-PLA nanoparticles, and studied the anti-inflammatory effect after administration [91]. In vivo experiment results showed that TP-PLA nanoparticles had a significant inhibitory effect on adjuvant—induced arthritis. Compatibility is a key characteristic of multi herb prescriptions. TCM formulations which contain two or more herbs can often result in better curative outcomes and fewer adverse effects than formulations with single herbs [92, 93]. Studies have shown that co-loaded nanocarrier systems could allow for the encapsulated drugs to exert a synergistic antitumor effect [94, 95]. Some studies have reported that the combination of curcumin and TP at low concentrations has a synergistic antitumor effect on ovarian cancer [96]. Liu et al. co-loaded TP and curcumin into nanoparticles prepared by mPEG-DPPE (TP/Curc-NPs). Through prescription screening, it was found that when TP and curcumin concentrations were 25.22 ng/mL and 6.62 g/mL, respectively, the synergistic killing effect of the two drugs on SKOV-3 tumor cells reached the maximum [97]. The synergistic anti-tumor mechanism of TP and Curc is mainly through the activation of caspase-3/9 and the inhibition of heat shock protein (HSP) expression to induce cellular apoptosis. After combined administration, TP/Curc-NPs reduced HSP70 mRNA levels while maintaining HSP90 mRNA levels. Since TP will produce excessive ROS in the liver and kidney, which leads to toxicity and damage to normal tissues, the combination of TP and curcumin can reduce ROS to achieve the purpose of attenuating toxicity and enhancing efficacy. In conclusion, TP/Curc-NPs has a good synergistic therapeutic effect, and curcumin can also alleviate the toxicity of TP to a certain extent. Therefore, TP/Curc-NPs may be a potential platform for ovarian cancer treatment. TP and celastrol (CL) are two monomers of TCM with various bioactivities isolated from TWHF. Silk fibroin protein is a kind of natural protein with many characteristics and is an ideal excipient for the preparation of nanoparticles. Ding et al. used SF as carrier material to prepare TP and CL loaded nanoparticles (TP-SFNPs and CL-SFNPs) respectively, and studied the synergistic therapeutic effect on pancreatic cancer cells (PC) [98]. Compared with free TP and CL, TP-SFNPs and CL-SFNPs can induce a large number of apoptosis of tumor cells due to the controlled release of TP and CL by SFNP during treatment. It showed good antitumor activity at the cellular level. The above results also reflect that the synergistic effect of TP-SFNPs and CL-SFNPs has a good therapeutic effect on PC.As one of the most widely used natural chemotherapy agents [99], paclitaxel (PTX) has long been a first-line drug for many cancers, such as non-small-cell lung cancer [100], glioblastoma [101], breast cancer [102], and However, long-term use causes cancer cells to develop resistance; thus, it has been reported that TP has anti-multidrug resistance in A549/Taxol cell lines mainly through inhibition of NF-κB signaling pathway and selective regulation of mitogen-activated protein kinase signaling pathway [103, 104]. Lipomeric hybrid nanoparticles (LPN) is a special nano carrier, which has the characteristics of both liposomes and polymerized nanoparticles [105, 106]. In view of this, in order to reduce drug resistance and then achieve the purpose of combined therapy, Liu et al. designed LPN as a combined drug delivery system of PTX and TP [107]. Compared with the control group, the nanoparticle group showed better anti-tumor effect. In vivo and in vitro experiments showed that PTX/TP-LPN had synergistic effects on lung cancer xenograft tumor, and the systemic toxicity was minimal. Although nanoparticles show great potential in TP delivery due to their controlled release of drugs, good biosafety and therapeutic effects [90, 91, 97], problems in mass production such as rigorous design and quality control still need to be thoroughly studied [108, 109].

#### Nanogels-based TP delivery

Hydrogels are three-dimensional network structures interwoven by hydrophilic polymers. Because of its good biocompatibility and drug delivery capacity, it is often injected directly into the site of the lesion for therapeutic purposes [110, 111]. In addition to preventing the drug from diffusing to normal tissues and causing side effects, it also has the effect of controlling the release and maintaining the concentration of the drug at the target site [112, 113] Considering the characteristics of hydrogels and nanoparticles, loading nanoparticles into the three-dimensional network structure of hydrogels can sustain drug release. This is one of the current main therapeutic strategies for hydrogels.

Due to the unique drug storage capacity of the hydrogel and the anti-inflammatory effect of TP, the combination of them can be used to treat RA and other diseases through transdermal administration. He et al. prepared the triptolide-loaded reduced graphene oxide hydrogel. Graphene nanosheets have good transdermal penetration while loading TP. Because of the strong π–π interaction between graphene oxide and triptolide, in vitro release studies showed that the release time of graphene hydrogels could be extended to 14 h (63.64–96.78%). The results of in vivo pharmacokinetic experiments showed that the relative bioavailability of graphene hydrogels was increased (3.3 fold) in comparison to the control hydrogels [114]. Analgesia and anti-inflammatory are two goals of RA treatment. Chen et al. cleverly brought them together using the gel as a platform [115]. In vitro and in vivo results showed that the pain threshold was increased and the inflammatory factors were effectively reduced after administration to the arthritis model rats.

In terms of anti-tumor therapy, Li et al. synthesized a triptolide-loaded injectable peptide hydrogel for in situ treatment of hepatocellular carcinoma [116]. In vitro results showed that the sustained release time of TP reached 14 days. Compared with human normal hepatocyte L-02, the liver cancer cell Bel-7402 has better cellular uptake and toxicity after administration. After in situ injection of nanogels into the tumor, TP was continuously released for more than 13 days and mainly accumulated in the target site, and the tumor inhibition rate was as high as 99.7%. Interesting, Zhang et al. designed a thermosensitive nano-hydrogel containing TP using a biodegradable material (poly (*N*-isopropylacrylamide). It is liquid at room temperature. After local injection into the tumor site, the gel is formed due to temperature rise, and TP released further mediates tumor apoptosis while anti-angiogenesis, which plays a good synergistic therapeutic effect [117].

Although nanohydrogels have the advantages of prolonged drug release, good biocompatibility, and biodegradability. However, for some special diseases that require drugs to quickly reach the plateau threshold, it is best to cooperate with other modes of administration.

### Stimuli-responsive nanoparticle-based TP delivery

Stimuli-responsive drug release and targeted drug delivery are two high-profile directions in cancer research, which have potential in intelligent and personalized cancer treatment [118]. In recent years, drug stimulus-responsive delivery systems have been extensively studied and have shown irreplaceable advantages in the diagnosis or treatment of various diseases. Researchers can design intelligent drug delivery systems based on physical or chemical factors, such as temperature, light, magnetism, hypoxia, glucose, pH, ultrasound, enzymes, and redox potential) and other lesion microenvironments or external interventions [119, 120]. Nanocarriers prepared using materials with stimuli-responsive release can deliver drugs to target sites or target cells and release them according to the responsive properties [121].

Due to the increase of ROS caused by chronic inflammation and the high expression of glutathione (GSH) caused by self-protection mechanisms, the microenvironment of tumor tissue is very different from that of normal tissue [122, 123]. Wang et al. used the special conditions of the tumor microenvironment to co-dissolve dithiodiacetic acid with PEG-2000-linoleic acid (mPEG2000-LD) in ethanol to develop a prodrug conjugated with TP and vitamin E (VE). In vitro release results showed that pegylated nano prodrug had a certain redox reaction, and the combination of PEGylated nano prodrug with TP prodrug had good targeting, sustained release and safety [27].

Based on the red fluorescence and good antitumor activity of doxorubicin (DOX), Wu et al. designed a stimuli-responsive release nano-drug delivery strategy [124]. Using a reduction-responsive polymer (mPEG-S–S-C16) and other excipients, a lipopolymer nanoparticle co-encapsulating DOX and TP was prepared. The results of in vitro drug release and cell internalization showed that the nanoparticles could successfully release the two drugs. In vitro and in vivo experiments showed that DOX/TP co-loaded LPNP (DOX/TP-LPNP) had a strong synergistic effect, and the combination index was low.

**Fig. 5:**
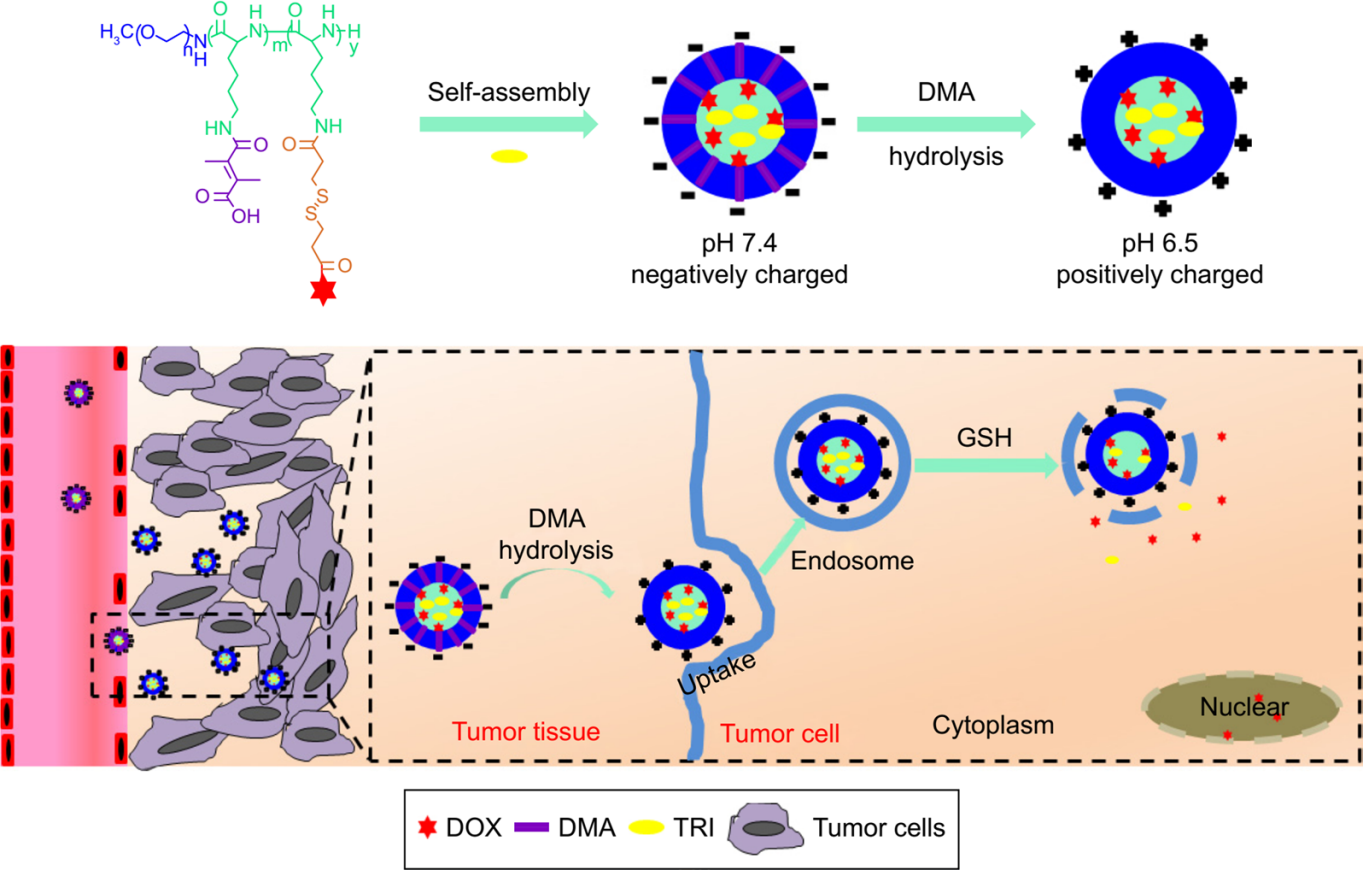
Schematic diagram of the structure and treatment of DA-SS-DT nanoparticles. Reproduced with permission from [128]

Negatively charged nanoparticle surfaces in the blood circulation have been reported to enhance stability, while conversion to positive charges in a slightly acidic tumor microenvironment enhances cellular uptake of the particles [125–127]. Based on this, Xu et al. designed a nanocarrier for pH-responsive charge switching and redox-responsive drug release [128]. The prepared nanoparticles were named DA-SS-DT. When DA-SS-DT reaches the tumor tissue due to the EPR effect after intravenous administration, the slightly acidic environment reversed its surface charge, which enhances the uptake of tumor cells. After cellular uptake, the drug begins to be released from the nanoparticles due to its redox-sensitive properties, which in turn exerts an antitumor effect (Fig. 5).

Stimulus-responsive drug delivery systems are multifunctional and smart; however, current developments in science and technology are not capable of mass production for clinical application. Therefore, a balance should be reached between application and research and development, striving to ensure that stimulus-responsive nanocarriers will eventually become practical in the near future.

### Active TDDS

Passive targeting utilizes the phenomenon of increased vascular permeability and decreased lymphatic return in tumor tissue to make nanoparticles accumulate in the tumor, namely EPR effect. Active targeting uses ligands modified on the surface of nanocarriers to bind to highly expressed receptors on the surface of target cells, followed by receptor-mediated endocytosis so that nanoparticles can selectively accumulate in target cells. The advantage of the active targeted drug delivery strategy is that in addition to increasing the concentration of the drug at the target site, it also reduces the accumulation of the drug in non-target organs, thereby reducing toxicity and enhancing efficacy [129–133].

**Fig. 6:**
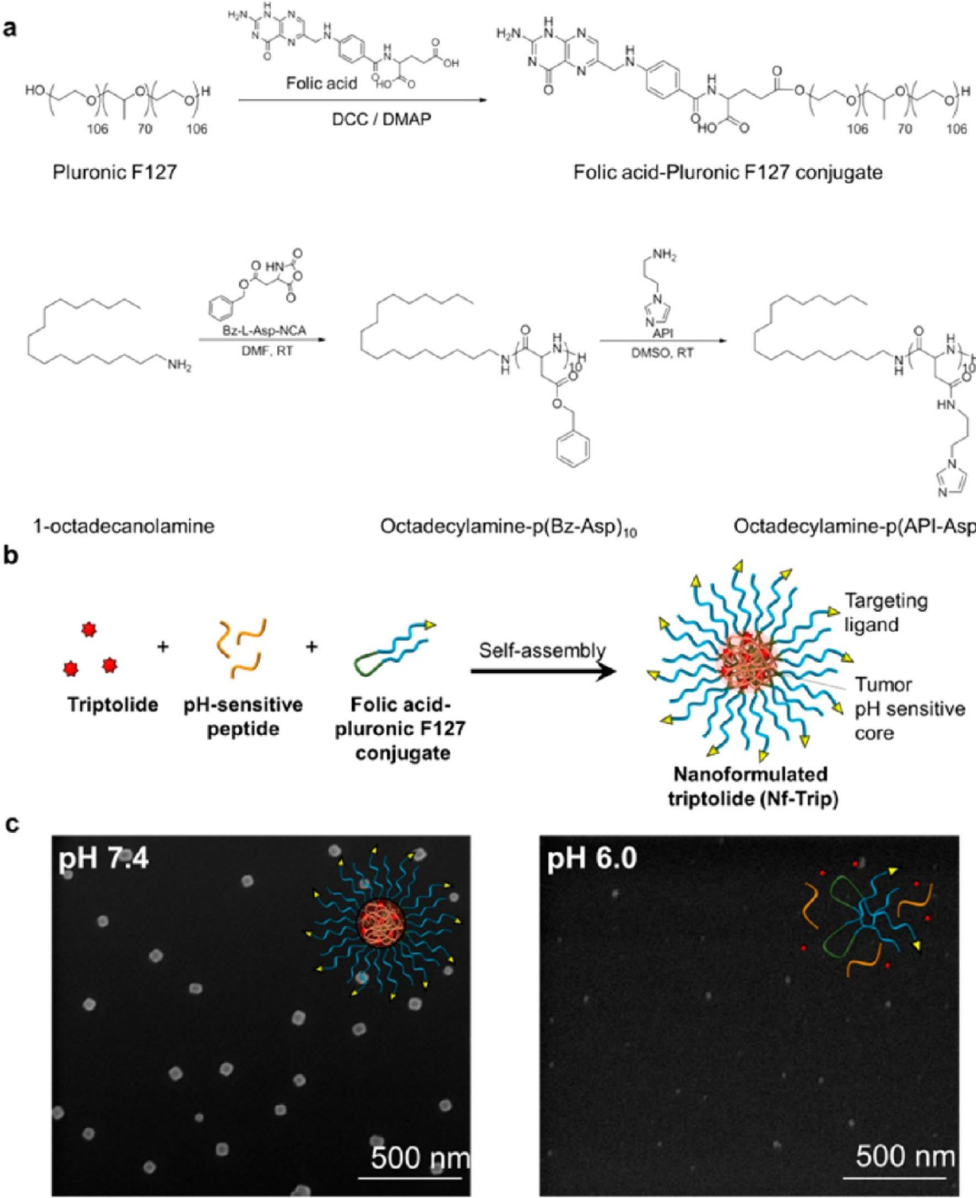
Synthesis and characterization of nano-formulated triptolide (Nf-Trip). **a** Synthetic scheme of Pluronic F127-folate conjugate and octadecylamine-p (API-Asp)10. **b** Fabrication of Nf-Trip through self-assembly. **c** SEM images of Nf-Trip at pH 7.4 and pH 6.0. Reproduced with permission from [138]

Because of its insensitivity and resistance to conventional chemotherapy drugs, HCC is one of the tumors with the worst prognosis [134, 135]. The combination of active targeting and stimulus reactivity does not only promote the specific accumulation of nanoparticles at the tumor site, but also improves the release of drugs at the target site, thereby improving therapeutic efficacy and reducing adverse effects [136, 137]. Through comparison and screening, Ling et al. found that the natural product TP has better antitumor activity than the current first-line drugs sorafenib, doxorubicin and daunorubicin. Considering some disadvantages of TP, the researchers synthesized tumor pH-sensitive TP nanoformulations containing folate for folate receptor (FA-R) overexpressed HCC [138]. The synthesis and characterization of nano-formulated TP (Nf-TP) are shown in Fig. 6. In vivo experiments indicated that the nanoparticles connected to folate ligands could specifically bind to FA-R on the surface of HCC, and receptor-mediated endocytosis resulted in the hydrolysis of the pH-responsive lipid core and the release of TP in cancer cells. The observed tumor inhibition effect was significantly better than that in the other groups, and the toxicity and adverse effects of TP were effectively controlled.

Unlike the FA-R on the surface of most solid tumor cells, the asialoglycoprotein receptor (ASGP-R) is specifically located on the surface of HCC cells [139, 140]. Aiming at this unique target, Zhang et al. developed a galactose-modified TP nanoparticle (GC-TP-NP), which can specifically target HCC for anti-tumor therapy. [141].

GC-TP-NP not only has a sustained release effect on drugs, but also galactose can specifically bind to ASGP-R, actively targeting liver cancer cells, improving drug efficacy and reducing systemic side effects. The researchers further studied and found that GC-TP-NP mainly induces cancer cell apoptosis by blocking the TNF-α/NF-κB/Bcl-2 signaling pathway. Compared with HCC, breast cancer is one of the most fatal cancers in women due to its rapid growth and rapid metastasis. In order to improve the tumor delivery rate and anti-metastatic effect of chemotherapy, Zhang et al. designed a CD44-targeting and pH/redox-sensitive nanosystem, which can actively target CD44 overexpressed on the cell surface through hyaluronic acid and release the drug in response to the tumor microenvironment, improving the antitumor effect. At the same time, lung metastasis of tumor was inhibited [142]. The results in vitro and in vivo indicated that the preparation had good effect on promoting tumor cell apoptosis and anti-tumor metastasis.

Acute kidney injury (AKI) is a kidney disease with high morbidity and mortality [143, 144]. Renal ischemia/reperfusion injury (Renal ischemia/reperfusion injury, IRI) is often caused after renal transplantation, cardiopulmonary resuscitation and aortic bypass surgery, trauma, hemorrhage, and is the main cause of AKI [145]. TP is an important bioactive compound with a variety of pharmacological activities, including the effective treatment of nephritis and renal IRI [146]. It is well known that the FA-R is highly expressed in tumors. However, FA-R is also commonly expressed in normal tissues and organs, such as the placenta, kidneys, and intestinal membranes [147].

To reduce toxicity and increase the effectiveness of TP, Huang et al. reported a biocompatible and high-efficiency renal-targeting nano-platform for IRI therapy, in which the toxic drug, TP, was encapsulated into FA-modified Pluronic F127/P123 nanoparticles (FPNP) [148].

**Fig. 7:**
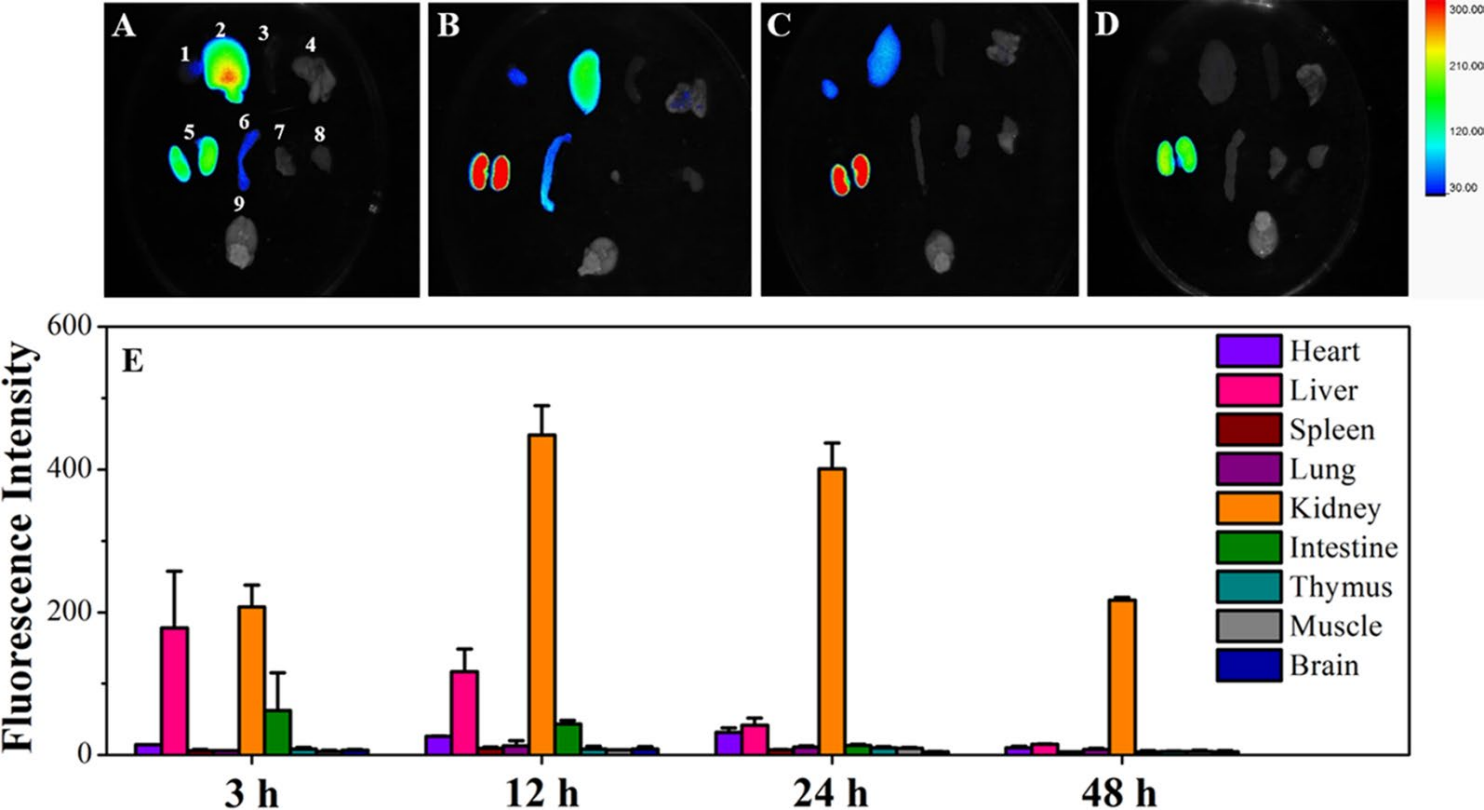
Ex vivo fluorescence images and biodistribution of Cy7 in organs (1–9: heart, liver, spleen, lung, kidneys, intestine, thymus, muscle, and brain) of mice after Cy7-FPNP injection at **A** 3, **B** 12, **C** 24, and **D** 48 h. **E** Corresponding fluorescence intensity of Cy7 distribution in each organ is examined (**A**–**D**). Data are expressed as mean ± SD (n = 3). Reproduced with permission from [148]

In vitro organ imaging results showed that FPNP had higher renal selectivity and longer retention time, as shown in Fig. 7. Systemic toxicity test showed that the nephrotoxicity, hepatotoxicity and reproductive system toxicity of TP-FPNP were significantly lower than those of free TP. Although there are many advantages of active targeted NDDS, the clinical application for disease treatment is very limited because of the following reasons: (1) the single target drug does not achieve the ideal targeting performance; (2) quality control of the complex system is difficult to carry out; and (3) high research and development costs lead to unaffordable medical bills. With the progress in science and technology, it is believed that there will be more stable, reliable, and low-cost active TDDS in clinical settings in the foreseeable future for the benefit of humankind.

The examples of TP combined with nano delivery system are shown in the Table 1, which are summarized and summarized according to nano-carrier, targeting category and treatment of type. The above summarizes the application of the combination of TP and TDDS in the medical field. Whether it is passive targeting, stimuli-responsive targeting or active targeting, all have their own advantages and characteristics. However, no one is perfect. In the selection of TDDS, we should make full use of the specific characteristics of diseases and drugs to avoid weaknesses. For example, some diseases require nanocarriers to quickly release drugs, some diseases require nanocarriers to have a sustained-release effect, and some diseases require nanocarriers to rapidly release drugs to the plateau threshold before sustained release. Therefore, for the problems currently faced, TDDS technology urgently needs to be upgraded and developed. There is still a long way to go in terms of safety and quality control for mass production, beyond the use of functional nanocarriers for therapeutic purposes.

**Table 1:** Summary of different delivery systems for triptolide

Nano-carriers	Targeting category	Treatment of type	Refs.
Polymeric micelles	Passive targeting	Anti-cancer	[53, 54, 57, 62]
Solid lipid nanoparticles	Passive targeting	Anti-inflammatory	[64, 68]
Liposomes	Redox-responsive	Anti-cancer	[76]
	Passive targeting	Anti-inflammatory	[77]
Polymeric nanoparticles	Passive targeting	Anti-cancer	[96–98, 107]
	Passive targeting	Anti-inflammatory	[90, 91]
	Redox-responsive	Anti-cancer	[27, 32, 124]
	Redox-responsive and pH-triggered charge-switchable	Anti-cancer	[128]
	Active targeting	Anti-cancer	[138, 141]
	Active targeting	Anti-renal ischemia/reperfusion injury	[148]
	Active targeting	Anti-cancer	[142]
Nanogels	Passive targeting	Anti-inflammatory	[114, 115]
	Thermo-responsive	Anti-cancer	[117]
	Passive targeting	Anti-cancer	[116]

## Conclusions

With a long history spanning over more than a thousand years, TCM still plays an irreplaceable role as an important part of human medical treatment. As a CHM, TP is a classic diterpenoid epoxide with a variety of pharmacological activities, such as anti-inflammatory, anti-autoimmunity, anti-cancer, and anti-fertility effects. Although TP has a good curative effect, its clinical application is limited by its severe toxicity, adverse effects, and poor water solubility. To reduce toxicity and increase efficacy, numerous drugs are delivered using advanced nano drug delivery systems. Based on the numerous examples of NDDS available in literature, this review systematically introduced passive and active targeting methods of TP administration, and preliminarily discussed their advantages and disadvantages. Whether using passive or active targeting, single or combined drug delivery approaches, the nano-targeted drug carriers containing TP have been reported to show site targeting, safety, and superior therapeutic effect compared with free TP. Moreover, stimulus-responsive drug carriers have demonstrated the advantage of intelligent drug delivery. Overall, these targeted delivery strategies could be used as a starting point for future utilization of TP and other TCMs in experimental therapy. Despite the limitations of NDDS and a large number of drug delivery systems are still in the pre-clinical research stage, especially for TCM preparations; however, advances in technology might prompt researchers to pay attention to the development of TCM into novel drug delivery systems.

## Data Availability

All data generated or analyzed during this study are included in this published article and the Additional Information.

## References

[1] Li LC, Zhang ZH, Zhou WC, Chen J, Jin HQ, Fang HM, Chen Q, Jin YC, Qu J, Kan LD (2020). Lianhua Qingwen prescription for Coronavirus disease 2019 (COVID-19) treatment: Advances and prospects. Biomed Pharmacother.

[2] Sun Z, He G, Huang N, Thilakavathy K, Lim JCW, Kumar SS, Xiong C (2021). Glycyrrhizic acid: a natural plant ingredient as a drug candidate to treat COVID-19. Front Pharmacol.

[3] Yang C, Pan X, Xu X, Cheng C, Huang Y, Li L, Jiang S, Xu W, Xiao G, Liu S (2020). Salvianolic acid C potently inhibits SARS-CoV-2 infection by blocking the formation of six-helix bundle core of spike protein. Signal Transduct Target Ther.

[4] Kupchan SM, Court WA, Dailey RG, Gilmore CJ, Bryan RF (1972). Triptolide and tripdiolide, novel antileukemic diterpenoid triepoxides from *Tripterygium wilfordii*. J Am Chem Soc.

[5] Zhou ZL, Yang YX, Ding J, Li YC, Miao ZH (2012). Triptolide: structural modifications, structure-activity relationships, bioactivities, clinical development and mechanisms. Nat Prod Rep.

[6] Alsaied OA, Sangwan V, Banerjee S, Krosch TC, Chugh R, Saluja A, Vickers SM, Jensen EH (2014). Sorafenib and triptolide as combination therapy for hepatocellular carcinoma. Surgery.

[7] Chen SR, Dai Y, Zhao J, Lin L, Wang Y, Wang Y (2018). A Mechanistic overview of triptolide and celastrol, natural products from *Tripterygium wilfordii* Hook F. Front Pharmacol.

[8] Li Y, Tian Y, Zhu W, Gong J, Li J (2013). Triptolide induces suppressor of cytokine signaling-3 expression and promotes lamina propria mononuclear cells apoptosis in Crohn's colitis. Int Immunopharmacol.

[9] Guan T, Shang W, Li H, Yang X, Fang C, Tian J, Wang K (2017). From detection to resection: photoacoustic tomography and surgery guidance with indocyanine green loaded gold Nanorod@liposome core-shell nanoparticles in liver cancer. Bioconjug Chem.

[10] Han R, Rostami-Yazdi M, Gerdes S, Mrowietz U (2012). Triptolide in the treatment of psoriasis and other immune-mediated inflammatory diseases. Br J Clin Pharmacol.

[11] Lü S, Wang Q, Li G, Sun S, Guo Y, Kuang H (2015). The treatment of rheumatoid arthritis using Chinese medicinal plants: from pharmacology to potential molecular mechanisms. J Ethnopharmacol.

[12] Wang J, Chu Y, Zhou X (2017). Inhibitory effect of *Triperygium wilfordii* polyglucoside on dipeptidyl peptidase I in vivo and in vitro. Biomed Pharmacother.

[13] Chang WT, Kang JJ, Lee KY, Wei K, Anderson E, Gotmare S, Ross JA, Rosen GD (2001). Triptolide and chemotherapy cooperate in tumor cell apoptosis. A role for the p53 pathway. J Biol Chem.

[14] Chen H, Zhou X, Gao Y, Zheng B, Tang F, Huang J (2014). Recent progress in development of new sonosensitizers for sonodynamic cancer therapy. Drug Discov Today.

[15] Meng C, Zhu H, Song H, Wang Z, Huang G, Li D, Ma Z (2014). Targets and molecular mechanisms of triptolide in cancer therapy. Chin J Cancer Res.

[16] Park K (2013). Triptolide induces apoptosis of PMA-treated THP-1 cells through activation of caspases, inhibition of NF-κB and activation of MAPKs. Int J Oncol.

[17] Shi JF, Luo YY, Li JX, Luo RF, Chen L, Li J, Zhang JM, Fu CM (2019). Research progress on anti-tumor effects and mechanisms of triptolide and its combined application. Zhongguo Zhong Yao Za Zhi.

[18] Carter BZ, Mak DH, Schober WD, McQueen T, Harris D, Estrov Z, Evans RL, Andreeff M (2006). Triptolide induces caspase-dependent cell death mediated via the mitochondrial pathway in leukemic cells. Blood.

[19] Tan BJ, Chiu GN (2013). Role of oxidative stress, endoplasmic reticulum stress and ERK activation in triptolide-induced apoptosis. Int J Oncol.

[20] Hong OY, Jang HY, Park KH, Jeong YJ, Kim JS, Chae HS (2021). Triptolide inhibits matrix metalloproteinase-9 expression and invasion of breast cancer cells through the inhibition of NF-κB and AP-1 signaling pathways. Oncol Lett.

[21] Liang M, Fu J (2008). Triptolide inhibits interferon-g-induced programmed death-1-ligand 1 surface expression in breast cancer cells. Cancer Lett.

[22] Zhang L, Yu JS (2019). Triptolide reverses helper T cell inhibition and down-regulates IFN-γ induced PD-L1 expression in glioma cell lines. J Neurooncol.

[23] Jiang X, Cao G, Gao G, Wang W, Zhao J, Gao C (2021). Triptolide decreases tumor-associated macrophages infiltration and M2 polarization to remodel colon cancer immune microenvironment via inhibiting tumor-derived CXCL12. J Cell Physiol.

[24] Li XJ, Jiang ZZ, Zhang LY (2014). Triptolide: progress on research in pharmacodynamics and toxicology. J Ethnopharmacol.

[25] Maeda H, Nakamura H, Fang J (2013). The EPR effect for macromolecular drug delivery to solid tumors: Improvement of tumor uptake, lowering of systemic toxicity, and distinct tumor imaging in vivo. Adv Drug Deliv Rev.

[26] Wen Y, Meng WS (2014). Recent in vivo evidences of particle-based delivery of small-interfering RNA (siRNA) into solid tumors. J Pharm Innov.

[27] Wang Y, Liu X, Wang X, Zheng W, Zhang J, Shi F, Liu J (2018). Redox-responsive self-assembly PEG nanoparticle enhanced triptolide for efficient antitumor treatment. Sci Rep.

[28] Zrazhevskiy P, Sena M, Gao X (2010). Designing multifunctional quantum dots for bioimaging, detection, and drug delivery. Chem Soc Rev.

[29] Hu X, Liu S (2015). Recent advances towards the fabrication and biomedical applications of responsive polymeric assemblies and nanoparticle hybrid superstructures. Dalton Trans.

[30] Tang L, Wang Z, Mu Q, Yu Z, Jacobson O, Li L, Yang W, Huang C, Kang F, Fan W (2020). Targeting neutrophils for enhanced cancer theranostics. Adv Mater.

[31] Tang L, Zhang F, Yu F, Sun W, Song M, Chen X, Zhang X, Sun X (2017). Croconaine nanoparticles with enhanced tumor accumulation for multimodality cancer theranostics. Biomaterials.

[32] He M, Yu L, Yang Y, Zou B, Li A (2020). Delivery of triptolide with reduction-sensitive polymer nanoparticles for liver cancer therapy on patient-derived xenografts models. Chin Chem Lett.

[33] Cheng L, Zhang X, Tang J, Lv Q, Liu J (2021). Gene-engineered exosomes-thermosensitive liposomes hybrid nanovesicles by the blockade of CD47 signal for combined photothermal therapy and cancer immunotherapy. Biomaterials.

[34] Dai Y, Sun Z, Zhao H, Qi D, Li X, Gao D, Li M, Fan Q, Shen Q, Huang W (2021). NIR-II fluorescence imaging guided tumor-specific NIR-II photothermal therapy enhanced by starvation mediated thermal sensitization strategy. Biomaterials.

[35] Nwabuife JC, Madhaorao Pant A, Govender T (2021). Liposomal delivery systems and their applications against *Staphylococcus aureus* and methicillin-resistant *Staphylococcus aureus*. Adv Drug Deliv Rev.

[36] Salunkhe SA, Chitkara D, Mahato RI, Mittal A (2021). Lipid based nanocarriers for effective drug delivery and treatment of diabetes associated liver fibrosis. Adv Drug Deliv Rev.

[37] Batty CJ, Bachelder EM, Ainslie KM (2021). Historical perspective of clinical nano and microparticle formulations for delivery of therapeutics. Trends Mol Med.

[38] Guo S, Wang Y, Miao L, Xu Z, Lin CH, Huang L (2014). Turning a water and oil insoluble cisplatin derivative into a nanoparticle formulation for cancer therapy. Biomaterials.

[39] Santos-Magalhães NS, Mosqueira VC (2010). Nanotechnology applied to the treatment of malaria. Adv Drug Deliv Rev.

[40] Shiokawa T, Hattori Y, Kawano K, Ohguchi Y, Kawakami H, Toma K, Maitani Y (2005). Effect of polyethylene glycol linker chain length of folate-linked microemulsions loading aclacinomycin A on targeting ability and antitumor effect in vitro and in vivo. Clin Cancer Res.

[41] Chen G, Yang Y, Xu Q, Ling M, Lin H, Ma W, Sun R, Xu Y, Liu X, Li N (2020). Self-amplification of tumor oxidative stress with degradable metallic complexes for synergistic cascade tumor therapy. Nano Lett.

[42] Du Y, Xia Y, Zou Y, Hu Y, Fu J, Wu J, Gao XD, Ma G (2019). Exploiting the lymph-node-amplifying effect for potent systemic and gastrointestinal immune responses via polymer/lipid nanoparticles. ACS Nano.

[43] Li R, Wang H, Song Y, Lin YN, Dong M, Shen Y, Khan S, Zhang S, Fan J, Zhang F F (2019). In situ production of Ag/polymer asymmetric nanoparticles via a powerful light-driven technique. J Am Chem Soc.

[44] Yang Y, Yu Y, Chen H, Meng X, Ma W, Yu M, Li Z, Li C, Liu H, Zhang X (2020). Illuminating platinum transportation while maximizing therapeutic efficacy by gold nanoclusters via Simultaneous near-infrared-I/II imaging and glutathione scavenging. ACS Nano.

[45] Bertrand N, Leroux JC (2012). The journey of a drug-carrier in the body: an anatomo-physiological perspective. J Control Release.

[46] Kotchey GP, Zhao Y, Kagan VE, Star A (2013). Peroxidase-mediated biodegradation of carbon nanotubes in vitro and in vivo. Adv Drug Deliv Rev.

[47] Ma W, Chen Q, Xu W, Yu M, Yang Y, Zou B, Zhang YS, Ding J, Yu Z (2021). Self-targeting visualizable hyaluronate nanogel for synchronized intracellular release of doxorubicin and cisplatin in combating multidrug-resistant breast cancer. Nano Res.

[48] Scott RC, Crabbe D, Krynska B, Ansari R, Kiani MF (2008). Aiming for the heart: targeted delivery of drugs to diseased cardiac tissue. Expert Opin Drug Deliv.

[49] Wen Y, Roudebush SL, Buckholtz GA, Goehring TR, Giannoukakis N, Gawalt ES, Meng WS (2014). Coassembly of amphiphilic peptide EAK16-II with histidinylated analogues and implications for functionalization of β-sheet fibrils in vivo. Biomaterials.

[50] Kawano K, Watanabe M, Yamamoto T, Yokoyama M, Opanasopit P, Okano T, Maitani Y (2006). Enhanced antitumor effect of camptothecin loaded in long-circulating polymeric micelles. J Control Release.

[51] Nishiyama N, Kataoka K (2006). Current state, achievements, and future prospects of polymeric micelles as nanocarriers for drug and gene delivery. Pharmacol Ther.

[52] Kedar U, Phutane P, Shidhaye S, Kadam V (2010). Advances in polymeric micelles for drug delivery and tumor targeting. Nanomedicine.

[53] Zheng S, Löw K, Wagner S, Yang X, von Briesen H, Zou S (2011). Cytotoxicity of triptolide and triptolide loaded polymeric micelles in vitro. Toxicol In Vitro.

[54] Xu L, Chen H, Xu H, Yang X (2008). Anti-tumour and immuno-modulation effects of triptolide-loaded polymeric micelles. Eur J Pharm Biopharm.

[55] Abdul Razak AR, Mau-Soerensen M, Gabrail NY, Gerecitano JF, Shields AF, Unger TJ, Saint-Martin JR, Carlson R, Landesman Y, McCauley D (2016). First-in-class, first-in-human phase I study of selinexor, a selective inhibitor of nuclear export, in patients with advanced solid tumors. J Clin Oncol.

[56] Mármol I, Sánchez-de-Diego C, Pradilla Dieste A, Cerrada E, Rodriguez Yoldi MJ (2017). Colorectal carcinoma: a general overview and future perspectives in colorectal cancer. Int J Mol Sci.

[57] Cui M, Jin M, Han M, Zang Y, Li C, Zhang D, Huang W, Gao Z, Yin X (2020). Improved antitumor outcomes for colon cancer using nanomicelles loaded with the novel antitumor agent LA67. Int J Nanomedicine.

[58] Abdollahi A, Folkman J (2010). Evading tumor evasion: current concepts and perspectives of anti-angiogenic cancer therapy. Drug Resist Updat.

[59] Takano S (2012). Glioblastoma angiogenesis: VEGF resistance solutions and new strategies based on molecular mechanisms of tumor vessel formation. Brain Tumor Pathol.

[60] He MF, Huang YH, Wu LW, Ge W, Shaw PC, But PP (2010). Triptolide functions as a potent angiogenesis inhibitor. Int J Cancer.

[61] Ma JX, Sun YL, Wang YQ, Wu HY, Jin J, Yu XF (2013). Triptolide induces apoptosis and inhibits the growth and angiogenesis of human pancreatic cancer cells by downregulating COX-2 and VEGF. Oncol Res.

[62] Wang C, Shan Y, Yang J, Xu X, Zhuang B, Fan Y, Xu W (2015). Inhibition of cancer angiogenesis using triptolide nanoparticles. J Biomed Nanotechnol.

[63] Mehnert W, Mäder K (2001). Solid lipid nanoparticles: production, characterization and applications. Adv Drug Deliv Rev.

[64] Mei Z, Li X, Wu Q, Hu S, Yang X (2005). The research on the anti-inflammatory activity and hepatotoxicity of triptolide-loaded solid lipid nanoparticle. Pharmacol Res.

[65] Luo H, Lu L, Liu N, Li Q, Yang X, Zhang Z (2021). Curcumin loaded sub-30 nm targeting therapeutic lipid nanoparticles for synergistically blocking nasopharyngeal cancer growth and metastasis. J Nanobiotechnol.

[66] Xue M, Zhao Y, Li XJ, Jiang ZZ, Zhang L, Liu SH, Li XM, Zhang LY, Yang SY (2012). Comparison of toxicokinetic and tissue distribution of triptolide-loaded solid lipid nanoparticles vs free triptolide in rats. Eur J Pharm Sci.

[67] Yaghmur A, Mu H (2021). Recent advances in drug delivery applications of cubosomes, hexosomes, and solid lipid nanoparticles. Acta Pharm Sin B.

[68] Gu Y, Yang M, Tang X, Wang T, Yang D, Zhai G, Liu J (2018). Lipid nanoparticles loading triptolide for transdermal delivery: mechanisms of penetration enhancement and transport properties. J Nanobiotechnol.

[69] Böttger R, Pauli G, Chao PH, Al Fayez N, Hohenwarter L, Li SD (2020). Lipid-based nanoparticle technologies for liver targeting. Adv Drug Deliv Rev.

[70] Torchilin V (2008). Antibody-modified liposomes for cancer chemotherapy. Expert Opin Drug Deliv.

[71] El-Samaligy MS, Afifi NN, Mahmoud EA (2006). Increasing bioavailability of silymarin using a buccal liposomal delivery system: preparation and experimental design investigation. Int J Pharm.

[72] Zhang Z, Mei L, Feng SS (2013). Paclitaxel drug delivery systems. Expert Opin Drug Deliv.

[73] Yi H, Lu W, Liu F, Zhang G, Xie F, Liu W, Wang L, Zhou W, Cheng Z (2021). ROS-responsive liposomes with NIR light-triggered doxorubicin release for combinatorial therapy of breast cancer. J Nanobiotechnol.

[74] El-Samaligy MS, Afifi NN, Mahmoud EA (2006). Evaluation of hybrid liposomes-encapsulated silymarin regarding physical stability and in vivo performance. Int J Pharm.

[75] Grimm JB, Tkachuk AN, Xie L, Choi H, Mohar B, Falco N, Schaefer K, Patel R, Zheng Q, Liu Z (2020). A general method to optimize and functionalize red-shifted rhodamine dyes. Nat Methods.

[76] Yu L, Wang Z, Mo Z, Zou B, Yang Y, Sun R, Ma W, Yu M, Zhang S, Yu Z (2021). Synergetic delivery of triptolide and Ce6 with light-activatable liposomes for efficient hepatocellular carcinoma therapy. Acta Pharm Sin B.

[77] Chen G, Hao B, Ju D, Liu M, Zhao H, Du Z, Xia J (2015). Pharmacokinetic and pharmacodynamic study of triptolide-loaded liposome hydrogel patch under microneedles on rats with collagen-induced arthritis. Acta Pharm Sin B.

[78] Feng L, Zhu C, Yuan H, Liu L, Lv F, Wang S (2013). Conjugated polymer nanoparticles: preparation, properties, functionalization and biological applications. Chem Soc Rev.

[79] Rao JP, Geckeler KE (2011). Polymer nanoparticles: preparation techniques and size-control parameters. Prog Polym Sci.

[80] Cai X, Wang KN, Ma W, Yang Y, Chen G, Fu H, Cui C, Yu Z, Wang X (2021). Multifunctional AIE iridium (III) photosensitizer nanoparticles for two-photon-activated imaging and mitochondria targeting photodynamic therapy. J Nanobiotechnol.

[81] Birk SE, Boisen A, Nielsen LH (2021). Polymeric nano- and microparticulate drug delivery systems for treatment of biofilms. Adv Drug Deliv Rev.

[82] Forier K, Raemdonck K, De Smedt SC, Demeester J, Coenye T, Braeckmans K (2014). Lipid and polymer nanoparticles for drug delivery to bacterial biofilms. J Control Release.

[83] Vauthier C, Bouchemal K (2009). Methods for the preparation and manufacture of polymeric nanoparticles. Pharm Res.

[84] Iannitelli A, Grande R, Di Stefano A, Di Giulio M, Sozio P, Bessa LJ, Laserra S, Paolini C, Protasi F, Cellini L (2011). Potential antibacterial activity of carvacrol-loaded poly(DL-lactide-co-glycolide) (PLGA) nanoparticles against microbial biofilm. Int J Mol Sci.

[85] Cheow WS, Chang MW, Hadinoto K (2011). The roles of lipid in anti-biofilm efficacy of lipid–polymer hybrid nanoparticles encapsulating antibiotics. Colloids Surf, A.

[86] Chávez de Paz LE, Resin A, Howard KA, Sutherland DS, Wejse PL (2011). Antimicrobial effect of chitosan nanoparticles on *Streptococcus mutans* biofilms. Appl Environ Microbiol.

[87] Cheow WS, Chang MW, Hadinoto K (2010). Antibacterial efficacy of inhalable antibiotic-encapsulated biodegradable polymeric nanoparticles against *E. coli* biofilm cells. J Biomed Nanotechnol.

[88] Chakraborty SP, Sahu SK, Pramanik P, Roy S (2012). In vitro antimicrobial activity of nanoconjugated vancomycin against drug resistant *Staphylococcus aureus*. Int J Pharm.

[89] Liu M, Dong J, Yang Y, Yang X, Xu H (2008). Effect of poly(d, l-lactic acid) nanoparticles as triptolide carrier on abating rats renal toxicity by NMR-based metabolic analysis. J Nanosci Nanotechnol.

[90] Zhang L, Chang J, Zhao Y, Xu H, Wang T, Li Q, Xing L, Huang J, Wang Y, Liang Q (2018). Fabrication of a triptolide-loaded and poly-γ-glutamic acid-based amphiphilic nanoparticle for the treatment of rheumatoid arthritis. Int J Nanomed.

[91] Liu M, Dong J, Yang Y, Yang X, Xu H (2005). Anti-inflammatory effects of triptolide loaded poly(d, l-lactic acid) nanoparticles on adjuvant-induced arthritis in rats. J Ethnopharmacol.

[92] Wang S, Hu Y, Tan W, Wu X, Chen R, Cao J, Chen M, Wang Y (2012). Compatibility art of traditional Chinese medicine: from the perspective of herb pairs. J Ethnopharmacol.

[93] Zhou M, Hong Y, Lin X, Shen L, Feng Y (2017). Recent pharmaceutical evidence on the compatibility rationality of traditional Chinese medicine. J Ethnopharmacol.

[94] Cressey P, Amrahli M, So PW, Gedroyc W, Wright M, Thanou M (2021). Image-guided thermosensitive liposomes for focused ultrasound enhanced co-delivery of carboplatin and SN-38 against triple negative breast cancer in mice. Biomaterials.

[95] Lang L, Shay C, Zhao X, Xiong Y, Wang X, Teng Y (2019). Simultaneously inactivating Src and AKT by saracatinib/capivasertib co-delivery nanoparticles to improve the efficacy of anti-Src therapy in head and neck squamous cell carcinoma. J Hematol Oncol.

[96] Cai YY, Lin WP, Li AP, Xu JY (2013). Combined effects of curcumin and triptolide on an ovarian cancer cell line. Asian Pac J Cancer Prev.

[97] Liu L, Xiong X, Shen M, Ru D, Gao P, Zhang X, Huang C, Sun Y, Li H, Duan Y (2018). Co-delivery of triptolide and curcumin for ovarian cancer targeting therapy via mPEG-DPPE/CaP nanoparticle. J Biomed Nanotechnol.

[98] Ding B, Wahid MA, Wang Z, Xie C, Thakkar A, Prabhu S, Wang J (2017). Triptolide and celastrol loaded silk fibroin nanoparticles show synergistic effect against human pancreatic cancer cells. Nanoscale.

[99] Rowinsky EK, Donehower RC (1995). Paclitaxel (taxol). N Engl J Med.

[100] Mazieres J, Kowalski D, Luft A, Vicente D, Tafreshi A, Gümüş M, Laktionov K, Hermes B, Cicin I, Rodríguez-Cid J (2020). Health-related quality of life with carboplatin-paclitaxel or nab-paclitaxel with or without pembrolizumab in patients with metastatic squamous non-small-cell lung cancer. J Clin Oncol.

[101] Ganipineni LP, Ucakar B, Joudiou N, Riva R, Jérôme C, Gallez B, Danhier F, Préat V (2019). Paclitaxel-loaded multifunctional nanoparticles for the targeted treatment of glioblastoma. J Drug Target.

[102] El-Azem N, Pulido-Moran M, Ramirez-Tortosa CL, Quiles JL, Cara FE, Sanchez-Rovira P, Granados-Principal S, Ramirez-Tortosa M (2019). Modulation by hydroxytyrosol of oxidative stress and antitumor activities of paclitaxel in breast cancer. Eur J Nutr.

[103] Jiang N, Dong XP, Zhang SL, You QY, Jiang XT, Zhao XG (2016). Triptolide reverses the Taxol resistance of lung adenocarcinoma by inhibiting the NF-κB signaling pathway and the expression of NF-κB-regulated drug-resistant genes. Mol Med Rep.

[104] Meng G, Wang W, Chai K, Yang S, Li F, Jiang K (2015). Combination treatment with triptolide and hydroxycamptothecin synergistically enhances apoptosis in A549 lung adenocarcinoma cells through PP2A-regulated ERK, p38 MAPKs and Akt signaling pathways. Int J Oncol.

[105] Hadinoto K, Sundaresan A, Cheow WS (2013). Lipid-polymer hybrid nanoparticles as a new generation therapeutic delivery platform: a review. Eur J Pharm Biopharm.

[106] Mandal B, Mittal NK, Balabathula P, Thoma LA, Wood GC (2016). Development and in vitro evaluation of core-shell type lipid-polymer hybrid nanoparticles for the delivery of erlotinib in non-small cell lung cancer. Eur J Pharm Sci.

[107] Liu J, Cheng H, Han L, Qiang Z, Zhang X, Gao W, Zhao K, Song Y (2018). Synergistic combination therapy of lung cancer using paclitaxel- and triptolide-coloaded lipid-polymer hybrid nanoparticles. Drug Des Devel Ther.

[108] Zhu ZJ, Wang H, Yan B, Zheng H, Jiang Y, Miranda OR, Rotello VM, Xing B, Vachet RW (2012). Effect of surface charge on the uptake and distribution of gold nanoparticles in four plant species. Environ Sci Technol.

[109] Zhu ZJ, Yeh YC, Tang R, Yan B, Tamayo J, Vachet RW, Rotello VM (2011). Stability of quantum dots in live cells. Nat Chem.

[110] Luo K, Wu H, Chen Y, Li J, Wang S (2021). Preparation of Bi-based hydrogel for multi-modal tumor therapy. Colloids Surf B: Biointerfaces.

[111] Jl A, Cz B, Jing ZA, Fei XC, Yz A, Swa C, Dz B (2021). Photo-induced tumor therapy using MnO 2/IrO 2-PVP nano-enzyme with TME-responsive behaviors. Colloids Surf B, Biointerfaces.

[112] Zhang YZC, Zhang Z, Zhao J, Yuan Y, Wang S (2021). Oxidation triggered formation of polydopamine-modified carboxymethyl cellulose hydrogel for anti-recurrence of tumor. Colloids Surf B: Biointerfaces.

[113] Di Tommaso L, Destro A, Seok JY, Balladore E, Terracciano L, Sangiovanni A, Iavarone M, Colombo M, Jang JJ, Yu E (2009). The application of markers (HSP70 GPC3 and GS) in liver biopsies is useful for detection of hepatocellular carcinoma. J Hepatol.

[114] Guo B, Qiao F, Liao Y, Song L, He J (2021). Triptolide laden reduced graphene oxide transdermal hydrogel to manage knee arthritis: in vitro and in vivo studies. J Biomater Sci Polym Ed.

[115] Ren S, Liu H, Wang X, Bi J, Lu S, Zhu C, Li H, Kong W, Chen R, Chen Z (2021). Acupoint nanocomposite hydrogel for simulation of acupuncture and targeted delivery of triptolide against rheumatoid arthritis. J Nanobiotechnol.

[116] Zhao X, Liu X, Zhang P, Liu Y, Ran W, Cai Y, Wang J, Zhai Y, Wang G, Ding Y, Li Y (2019). Injectable peptide hydrogel as intraperitoneal triptolide depot for the treatment of orthotopic hepatocellular carcinoma. Acta Pharm Sin B.

[117] Luo Y, Li J, Hu Y, Gao F, Pak-Heng Leung G, Geng F, Fu C, Zhang J (2020). Injectable thermo-responsive nano-hydrogel loading triptolide for the anti-breast cancer enhancement via localized treatment based on "two strikes" effects. Acta Pharm Sin B.

[118] Daglioglu C (2018). Environmentally responsive dual-targeting nanoparticles: improving drug accumulation in cancer cells as a way of preventing anticancer drug efflux. J Pharm Sci.

[119] Yang C, Pang X, Chen W, Wang X, Liu G (2019). Environmentally responsive dual-targeting nanotheranostics for overcoming cancer multidrug resistance. Sci Bull.

[120] Huang B, Chen F, Shen Y, Qian K, Wang Y, Sun C, Zhao X, Cui B, Gao F, Zeng Z, Cui H (2018). Advances in targeted pesticides with environmentally responsive controlled release by nanotechnology. Nanomaterials.

[121] Bruschi ML, Borghi-Pangoni FB, Junqueira MV, de Souza Ferreira SB, da Silva JB (2017). Environmentally responsive systems for drug delivery. Recent Pat Drug Deliv Formul.

[122] Xu Z, Liu S, Kang Y, Wang M (2015). Glutathione- and pH-responsive nonporous silica prodrug nanoparticles for controlled release and cancer therapy. Nanoscale.

[123] Guo D, Huang Y, Jin X, Zhang C, Zhu X (2021). A redox-responsive, in-situ polymerized polyplatinum(IV)-coated gold nanorod as an amplifier of tumor accumulation for enhanced thermo-chemotherapy. Biomaterials.

[124] Wu B, Lu ST, Zhang LJ, Zhuo RX, Xu HB, Huang SW (2017). Codelivery of doxorubicin and triptolide with reduction-sensitive lipid-polymer hybrid nanoparticles for in vitro and in vivo synergistic cancer treatment. Int J Nanomed.

[125] Chen S, Rong L, Lei Q, Cao PX, Qin SY, Zheng DW, Jia HZ, Zhu JY, Cheng SX, Zhuo RX, Zhang XZ (2016). A surface charge-switchable and folate modified system for co-delivery of proapoptosis peptide and p53 plasmid in cancer therapy. Biomaterials.

[126] You-Yong Y, Cheng-Qiong M, Xiao-Jiao Du, Jin-Zhi Du, Feng W (2012). Surface charge switchable nanoparticles based on zwitterionic polymer for enhanced drug delivery to tumor. Adv Mater.

[127] Yue W, Lv S, Deng M, Tang Z, Chen X (2016). A charge-conversional intracellular-activated polymeric prodrug for tumor therapy. Polym Chem.

[128] Xu C, Song RJ, Lu P, Chen JC, Zhou YQ, Shen G, Jiang MJ, Zhang W (2018). pH-triggered charge-reversal and redox-sensitive drug-release polymer micelles codeliver doxorubicin and triptolide for prostate tumor therapy. Int J Nanomed.

[129] Li Y, Xiao Y, Lin HP, Reichel D, Bae Y, Lee EY, Jiang Y, Huang X, Yang C, Wang Z (2019). In vivo β-catenin attenuation by the integrin α5-targeting nano-delivery strategy suppresses triple negative breast cancer stemness and metastasis. Biomaterials.

[130] Satsangi A, Roy SS, Satsangi RK, Tolcher AW, Vadlamudi RK, Goins B, Ong JL (2015). Synthesis of a novel, sequentially active-targeted drug delivery nanoplatform for breast cancer therapy. Biomaterials.

[131] Zhang H, Liu J, Chen Q, Mi P (2020). Ligand-installed anti-VEGF genomic nanocarriers for effective gene therapy of primary and metastatic tumors. J Control Release.

[132] Li J, Zeng H, You Y, Wang R, Tan T, Wang W, Yin L, Zeng Z, Zeng Y, Xie T (2021). Active targeting of orthotopic glioma using biomimetic liposomes co-loaded elemene and cabazitaxel modified by transferritin. J Nanobiotechnol.

[133] Zhang P, Zhang L, Qin Z, Hua S, Guo Z, Chu C, Lin H, Zhang Y, Li W, Zhang X (2018). Genetically engineered liposome-like nanovesicles as active targeted transport platform. Adv Mater.

[134] Llovet JM, De Baere T, Kulik L, Haber PK, Greten TF, Meyer T, Lencioni R (2021). Locoregional therapies in the era of molecular and immune treatments for hepatocellular carcinoma. Nat Rev Gastroenterol Hepatol.

[135] Maluccio M, Covey A (2012). Recent progress in understanding, diagnosing, and treating hepatocellular carcinoma. CA Cancer J Clin.

[136] Wang J, Dong Y, Li Y, Li W, Cheng K, Qian Y, Xu G, Zhang X, Hu L, Chen P (2018). Designer exosomes for active targeted chemo-photothermal synergistic tumor therapy. Adv Func Mater.

[137] Zhang P, Wang C, Zhao J, Xiao A, Shen Q, Li L, Li J, Zhang J, Min Q, Chen J (2016). Near infrared-guided smart nanocarriers for microRNA-controlled release of doxorubicin/siRNA with intracellular ATP as fuel. ACS Nano.

[138] Ling D, Xia H, Park W, Hackett MJ, Song C, Na K, Hui KM, Hyeon T (2014). pH-sensitive nanoformulated triptolide as a targeted therapeutic strategy for hepatocellular carcinoma. ACS Nano.

[139] Alex SM, Rekha MR, Sharma CP (2011). Spermine grafted galactosylated chitosan for improved nanoparticle mediated gene delivery. Int J Pharm.

[140] Zheng D, Duan C, Zhang D, Jia L, Liu G, Liu Y, Wang F, Li C, Guo H, Zhang Q (2012). Galactosylated chitosan nanoparticles for hepatocyte-targeted delivery of oridonin. Int J Pharm.

[141] Zhang YQ, Shen Y, Liao MM, Mao X, Mi GJ, You C, Guo QY, Li WJ, Wang XY, Lin N, Webster TJ (2019). Galactosylated chitosan triptolide nanoparticles for overcoming hepatocellular carcinoma: enhanced therapeutic efficacy, low toxicity, and validated network regulatory mechanisms. Nanomedicine.

[142] Shi J, Ren Y, Ma J, Luo X, Li J, Wu Y, Gu H, Fu C, Cao Z, Zhang J (2021). Novel CD44-targeting and pH/redox-dual-stimuli-responsive core-shell nanoparticles loading triptolide combats breast cancer growth and lung metastasis. J Nanobiotechnol.

[143] Kang R, Rovin B (2018). Advances and challenges on new therapies and clinical targets of acute kidney injury. Toxicol Pathol.

[144] Sharp CN, Siskind LJ (2017). Developing better mouse models to study cisplatin-induced kidney injury. Am J Physiol Renal Physiol.

[145] Chertow GM, Burdick E, Honour M, Bonventre JV, Bates DW (2005). Acute kidney injury, mortality, length of stay, and costs in hospitalized patients. J Am Soc Nephrol.

[146] Qi B, Wang X, Zhou Y, Han Q, He L, Gong T, Sun X, Fu Y, Zhang Z (2015). A renal-targeted triptolide aminoglycoside (TPAG) conjugate for lowering systemic toxicities of triptolide. Fitoterapia.

[147] Chen C, Ke J, Zhou XE, Yi W, Brunzelle JS, Li J, Yong EL, Xu HE, Melcher K (2013). Structural basis for molecular recognition of folic acid by folate receptors. Nature.

[148] Huang C, Zeng T, Li J, Tan L, Deng X, Pan Y, Chen Q, Li A, Hu J (2019). Folate receptor-mediated renal-targeting nanoplatform for the specific delivery of triptolide to treat renal ischemia/reperfusion injury. ACS Biomater Sci Eng.

